# Moderate Coconut Oil Supplement Ameliorates Growth Performance and Ruminal Fermentation in Hainan Black Goat Kids

**DOI:** 10.3389/fvets.2020.622259

**Published:** 2020-12-23

**Authors:** Liguang Shi, Yu Zhang, Lingli Wu, Wenjuan Xun, Qiang Liu, Ting Cao, Guanyu Hou, Hanlin Zhou

**Affiliations:** ^1^Tropical Crops Genetic Resources Institute, Chinese Academy of Tropical Agricultural Sciences, Haikou, China; ^2^College of Animal Science and Veterinary Medicine, Shanxi Agricultural University, Taigu, China; ^3^College of Animal Sciences and Technology, Hainan University, Haikou, China

**Keywords:** coconut oil, growth performance, nutrient digestibility, ruminal fermentation, blood metabolites

## Abstract

The study investigated amelioration effects of coconut oil (CO) on growth performance, nutrient digestibility, ruminal fermentation, and blood metabolites in Hainan Black goat kids. Twenty-four Hainan Black goat kids (10 days of age) were assigned randomly to four treatments for 90 days, including pre-weaning (10–70 d of age) and post-weaning (70-100 d of age) days. The treatment regimens were control (CON), low CO (LCO), medium CO (MCO), and high CO (HCO) with 0, 4, 6, 8 g CO per goat per day, respectively. During the pre-weaning period, the average daily gain (ADG) linearly and quadratically increased (*P* < 0.05), whereas the average daily feed intake (ADFI) linearly decreased, and the feed conversion ratio (FCR) also decreased linearly and quadratically by increasing CO supplementation (*P* < 0.05). During the post-weaning period, increasing CO supplementation linearly and quadratically increased the BW at 100 days and ADG (*P* < 0.05), but quadratically decreased the ADFI and FCR (*P* < 0.05). The digestibility of ether extract (EE) linearly and quadratically increased with increasing CO supplementation (*P* < 0.05). Supplementation of CO linearly increased ruminal pH (P < 0.05), but linearly decreased (*P* < 0.05) ammonia-N, total VFAs, molar proportions of acetate, ruminal microbial enzyme activity of carboxymethyl-cellulase, cellobiase, xylanase, pectinase and α-amylase, and number of total protozoa, the abundance of *Ruminococcus albus, Ruminococcus flavefaciens, Fibrobacter succinogenes, Butyrivibrio fibrisolvens, Prevotella ruminicola*, and *Ruminobacter amylophilus*. The estimated methane emission decreased linearly and quadratically with increasing CO addition (*P* < 0.05). The serum concentration of triglycerides (TG), non-esterified fatty acids (NEFA) and growth hormone (GH) linearly (*P* < 0.05) increased by raising the CO supplementation. The present results indicate that CO supplementation at 6 g/day per goats is optimum due to improved growth performance and decreased estimated methane emission. Supplementation CO up to 8 g/day depressed growth and feed conversion due to its suppression of growth performance, rumen protozoa, cellulolytic bacteria and microbial enzyme activity, and reduced ADF and ADF digestibility.

## Introduction

Goats are important meat-producing animals and goat meat is well-appreciated by consumers worldwide, especially in developing countries (1). Hainan Black goats are the main goat breed in South China, characterized by a good adaptability to the local hot and wet weather (2). Hainan Black goat meat is also very popular in South China because of its delicious flavors. However, Hainan Black goats exhibit slow growth rates and small body sizes, resulting in poor carcass characteristics (3).

Manipulation of the rumen microbial ecosystem to enhance fiber digestion, reduce the excretion of methane and urea, in order to improve the production performance of ruminants is one of the most important goals for animal nutritionists (4, 5). Dietary fats have been used to improve ruminant growth performance and modify meat characteristics with human health benefits (6). Moreover, fat supplementation in the diet of newborn lambs would be considered an effective mechanism to modify the rumen microbiome (7, 8). Therefore, further understanding of the effects of fat on rumen fermentation may help to offer a nutritional strategy to reduce rumen methane emissions and improve the quality of ruminant products.

Among all the lipid feedstocks, vegetable oils, oilseed, and calcium salts of fatty acids are the most appropriate for application in ruminant diets (9). Coconut oil is a cheaper, tastier, and readily available feed resource for ruminants (10). Coconut oil is a highly saturated oil (About 90% saturation), which is rich in medium chain fatty acids (MCFAs) (11). MCFA have been reported to reduce fat deposition due to their faster metabolism and reduced storage in adipocytes (12). Furthermore, coconut oil has been proven to exert positive environmental effects by enhancing rumen fermentation via limiting the production of methane and modifying microbial populations (13–15). There have been discrepancies in the results obtained by studies conducted to evaluate the effects of coconut oil supplementation on nutrient digestibility, growth performance and body composition of ruminants. The studies reported by Ding et al. (16) found that supplementing 12 g CO or 0.48 g/kg BW daily, showed a strong methane reduction as well as a decrease in the number of methanogen and *Fibrobacter succinogenes* in Tibetan sheep. Similar effects were also observed by Liu et al. (17) who reported that supplementation with 0.52 g/kg BW CO in sheep decreased methane emissions by reducing the methanogen and protozoa populations without negatively affecting the growth performance or reduction of rumen total VFA. Besides, the anti-methane effects of CO were also observed in swamp buffalo (18) and dairy cows (19), and neither study identified negative effects of CO on DMI, nutrient digestibility or ruminal fermentation. However, a study on beef heifers with different levels of CO demonstrated a linear decrease in CH_4_ production without affecting the DMI or giving rise to negative effects on DMI and digestibility at lower doses, with only the highest dose of 375 g/d yielding undesirable effects on the DMI and digestibility (20). Another study in lambs revealed that CO supplementation at 50 g/kg in the concentrate improved the feed conversion ratio and carcass traits of lambs, but its higher inclusion in ruminant diets has negative effects on growth and feed conversion due to its depressing impact on rumen protozoa which results in lower fiber digestibility (10). We hypothesized that in ruminant species, the level of fat, and the nature of the basal diet may determine the variable effects of CO on ruminal microbes.

Considering the inconsistent results regarding the impact of CO supplementation on growth performance, nutrient digestibility, and ruminal fermentation, as well as the limited research performed in goat kids, this study was undertaken to investigate the effects of coconut oil on growth performance, nutrient digestion, ruminal fermentation, and blood metabolites in Hainan Black goat kids.

## Materials and Methods

### Animals and Experimental Design

The animal and experiment protocols were approved by the Animal Care and Use Committee of Chinese Academy of Tropical Agricultural Sciences (ACUCC), Hainan, PR China. Twenty-four Hainan Black goat kids averaging 10 days of age and 2.05 ± 0.16 kg of body weight (BW) were randomly assigned to four treatment regimens. The treatments consisted of control (CON), low CO (LCO), medium CO (MCO) and high CO (HCO) dosages containing 0, 4, 6, 8 g of CO per goat daily, respectively. The CO supplement was purchased commercially and sprayed into the back of the kids' mouth using a small syringe, twice a day at 0700 and 1700 h throughout the experimental period. From 10 to 70 days of age (weaning), the goat kids were fed with a milk replacer (2% of BW) twice a day at 0800 h and 1800 h for 30 days, after which the daily milk portion was decreased by half until weaning. The goats were weighed weekly to calculate the amount of milk replacer to be administered. The goats were also offered an *ad libitum* concentrate and dried king grass in a cafeteria system during the whole experimental period, and the dietary concentrate to forage ratio was maintained at 50:50 based on an air-dry matter. All goats were fed the same concentrate mixture. The post-weaning feeding management for all goats was kept identical that of the pre-weaning phase, except for the fact that administration of the milk replacer stopped at 70 days of age. The ingredients and chemical composition of the experimental diets were illustrated in Table 1. Fresh water was available to the goats for drinking throughout the experimental period. The animals were weighed at 10, 70, and 100 days of age before feeding, and the average daily gain (ADG) was recorded.

**Table 1:** Ingredient and chemical composition of basal diets (Air-dry matter basis).

**Item**	**Content**
**Ingredients of diet (%)**	
Dried king grass	50.00
Corn	34.00
Soybean meal	9.00
Wheat bran	4.90
Shell powder	0.70
Sodium bicarbonate	0.30
Salt	0.70
Calcium carbonate	0.40
**Chemical composition of diet**	
Organic matter (%)	93.34
Crude protein (%)	16.55
Ether extract (%)	2.64
Neutral detergent fiber (%)	39.63
Acid detergent fiber (%)	26.45
Calcium (%)	0.32
Phosphorus (%)	0.22
Gross energy (MJ/kg)	18.12

### Data Collection and Sampling Procedures

The milk intake of individual goats was measured during the pre-weaning period. Feed offered and refusals for each goat were also recorded on a daily basis throughout the experimental period so as to calculate the daily DM intake (DMI). The goats were dosed via the esophagus with 1 g of chromic oxide in a paper capsule twice daily (07:00 and 19:00 h) from 78–87 days of age. The chromic oxide powder was used as a digestion marker to estimate the fecal excretion. From 83–87 days of age, Fecal pellets were collected from the rectum at 7:00, 15:00, and 24:00, then representative samples of the feces were pooled. The samples of feeds, refusals and feces were pooled for each goat, dried at 60°C for 48 h, ground to pass a 1 mm sieve, and preserved for chemical composition analysis. The apparent nutrient digestibility was calculated according to our prior studies (21).

Samples of rumen fluid were collected using an oral stomach tube at 07:00 by 70 days of age. The initial 100 mL ruminal fluid extracted was discarded, and the next 100 mL was retained. The fluid's pH values were immediately measured using a pH meter (PHS-3C, Shanghai Leijun experimental instrument Co., Ltd., Shanghai, China). After pH measurement, the rumen fluid was filtered through four layers of cheesecloth and subsampled for various determinations. A 5 ml filtrate was preserved by adding 1 mL of 250 g/L meta-phosphoric acid or 1 mL of 20 g/L H_2_SO_4_ to determine the VFA and NH_3_ concentrations, respectively. These samples were then frozen at −20°C until further analysis. About 50 mL of filtrate was collected and frozen at −80°C for DNA extraction, and another 40 mL of filtrate was used to determine the activity of ruminal enzymes according to the method described by Agarwal (22).

At 70 and 100 days of age, about 5 ml of blood was collected from the jugular vein and harvested into tubes without anticoagulant before the morning feeding at 100 days of age. Serum samples were then centrifuged at 3,000 × g for 15 min at 4°C and stored at −20°C until the assay.

### Chemical Analyses

Oven-dried samples were analyzed for DM method 934.01), OM (method 942.05), nitrogen (method 976.05), ether extract (method 973.18) and acid detergent fiber (ADF; method 973.18) according to AOAC methods (23). The neutral detergent fiber (aNDF) was analyzed using methods described by Van Soest et al. (24) with heat stable alpha amylase and sodium sulfite utilized in the NDF procedure, and results were expressed inclusive of residual ash. Ruminal VFA concentration was measured by gas chromatography (HP Agilent 6890N, Santa Clara, CA, USA) with a flame ionization detector equipped with an HP-INNOWAX (19091N-133) capillary column (30 m × 0.25 mm × 0.25 μm). Two microliter of fluid samples were injected with a syringe, and the injector and detector temperature were programmed at 200 and 220°C, respectively. Nitrogen was used as a carrier flowing at 5.5 mL/min. A program altered oven temperature from 80 to 170°C at 15°C/min and then held it at 170°C for 1.5 min. Ruminal VFA were expressed on the basis of absolute concentrations (mM) and molar proportions (mol/100 mol total VFA). Ruminal ammonia-N concentration was determined by a colorimetric spectrophotometer (UV2100, Shanghai Younike instrument Co., Ltd., Shanghai, China) according to AOAC methods (2000). Subsequently ruminal fluid samples were sonicated at 4°C in an ice bath with a 30 s pulsation rate for 10 min, then centrifuged at 3,000 × g at 4°C for 20 min. The resulting supernatant was used for estimation of the enzyme activity (carboxymethyle cellulase, cellobiase, xylanase, pectinase, α-amylase and protease) as described by Agarwal et al. (22). Serum parameters including glucose, cholesterol, and triglycerides were determined by using the BH13 MD 1600 (America) automatic biochemical analyzer. Serum level of non-esterified fatty acids (Nanjing Jiancheng Bioengineering Institute, Nanjing, China) and growth hormone (Shanghai Fankel Industrial Co., Ltd, Shanghai, China) were determined by using enzyme-linked immunosorbent assay (ELISA) kits according to the manufacturer's instructions.

### DNA Extraction and Quantitative Real-Time PCR

Microbial DNA was extracted from 0.5 g of rumen fluid by using a Fastpure Bacteria DNA Isolation Mini Kit (Vazyme, Version 8.1). Subsequently, agarose gel electrophoresis and the NanoDrop 2000 Spectrophotometer (NanoDrop Technologies, USA), were used to evaluate the quality and quantity of DNA, respectively. The extracted DNA was then kept frozen at −20°C for real time PCR analysis. Populations of *Ruminococcus albus, Ruminococcus flavefaciens, F. succinogenes, Butyrivibrio fibrisolvens, Prevotella ruminicola*, and *Ruminobacter amylophilus* were estimated using real time PCR as a proportion of the total number of bacteria. The sequences of all primers were synthesized by Tianyi Huiyuan Biotechnology Co., Ltd and displayed in Table 2. All real-time PCR reactions were carried out in triplicate and run on Applied Biosystems 7500 Fast real-time quantitative PCR systems. The reaction mixture (20 μL) contained 10 μL SYBR Color qPCR Master Mix (Vazyme Biotechnology Co., Ltd., Nanjing, China), 0.4 μL 10 μmol/L PCR Forward Primer, 0.4 μL 10 μmol/L PCR Reverse Primer, 0.4 μL ROX Reference Dye (50×), 6.8 μL ddH_2_O and 2 μL of the template DNA. The quantity of DNA was measured in triplicate for each sample using the ND-1000 UV spectrophotometer (NanoDrop Technologies, USA), and the mean values were estimated. PCR was implemented according to the following conditions: Degeneration at 95°C for 60 s; PCR reaction at 95°C for 15 s and 60°C for 30 s, with 40 cycles; dissociation stage.

**Table 2:** The primer of ruminal bacteria and 16s rRNA genes.

**Target species**	**Primer sequence**	**GeneBank accession no**.	**Size (bp)**
Total bacteria	F: CGGCAACGAGCGCAACCC	AY548787.1	147
	R: CCATTGTAGCACGTGTGTAGCC		
Total protozoa	F: GCTTTCGWTGGTAGT GTATT	HM212038.1	234
	R: CTTGCCCTCYAATCGTWCT		
*R. albus*	F: CCCTAAAAGCAGTCTTAGTTC G	CP002403.1	175
	R: CCTCCTTGCGGTTAGAACA		
*R. flavefaciens*	F: ATTGTCCCAGTTCAGATTGC	AB849343.1	132
	R: GGCGTCCTCATTGCTGTTAG		
*F. succinogenes*	F: GTTCGGAATTACTGGGCGTAA A	AB275512.1	121
	R: CGCCTGCCCCTGAACTATC		
*B. fibrisolvens*	F: ACCGCATAAGCGCACGGA	HQ404372.1	65
	R: CGGGTCCATCTTGTACCGATA AAT		
*R. amylophilus*	F: CTGGGGAGCTGCCTGAATG	MH708240.1	102
	R: GCATCTGAATGCGACTGGTTG		
*P. ruminicola*	F: GAAAGTCGGATTAATGCTCTATGTTG	LT975683.1	74
	R: CATCCTATAGCGGTAAACCTTTGG		

### Statistical Analyses

Data analysis was conducted using the SAS mixed model procedure (Proc Mixed; SAS, 2002). Analysis of variance (ANOVA) was performed to examine the effects of the respective treatment regimens on growth performance, nutrient digestibility, ruminal fermentation, and blood metabolites. Linear and quadratic effects were tested using the CONTRAST statement of SAS with coefficients estimated based on the CON application rates. Differences between the treatment regimens were detected by the Duncan's multiple range test. The *P*-value for statistical significance was set at *P* ≤ 0.05, unless otherwise noted *P* ≤ 0.10 was considered as a tendency approaching significance.

## Results

### Dry Matter Intake, Average Daily Gain, and Feed Conversion Ratio

Dry matter intake, average daily gain and feed conversion ratio were delineated in Table 3. The dry matter intake (DMI) exhibited a linear decline (*P* < 0.05) with increasing CO supplementation for pre-weaned and post-weaned goats, and was lower for HCO than for control, LCO, and MCO (*P* < 0.05). Meanwhile the average daily gain (ADG) for pre-weaned and post-weaned goats increased linearly (*P* < 0.05) and quadratically (*P* < 0.05) with increasing CO supplementation and was higher for MCO than that for control, LCO, and HCO (*P* < 0.05). The feed conversion ratio (FCR) for pre-weaned and post-weaned goats decreased linearly (*P* < 0.05) and quadratically (*P* < 0.05) with increasing CO supplementation, and was lower for MCO group than control and HCO (*P* < 0.05).

**Table 3:** Effects of coconut oil on dry matter intake, average daily gain and feed conversion ratio in goat kids.

	**Treatment^e^**					***P-*****value^f^**		
**Item^f^**	**CON**	**LCO**	**MCO**	**HCO**	**SEM^g^**	**Treatment**	**Linear**	**Quadratic**
**Pre-weaning (10~70 days of age)**								
**Body weight (kg)**								
Birth weight	1.82	1.91	1.93	1.87	0.157	0.982	0.921	0.775
10 days	2.05	2.13	2.17	2.03	0.086	0.994	0.956	0.786
70 days	5.57^b^	6.23^a^^b^	7.57^a^	6.10^a^^b^	0.249	0.020	0.134	0.020
ADG (g/d)	60.15^b^	69.47^b^	91.12^a^	68.30^b^	2.808	0.001	0.004	0.001
DMI (g/d)	325.17^a^	325.50^a^	323.17^a^	309.33^b^	2.184	0.014	0.006	0.152
FCR (kg/kg)	5.47^a^	4.70^b^	3.56^c^	4.62^b^	0.176	0.001	0.001	0.001
**Post-weaning (70~100 days of age)**								
**Body weight (kg)**								
100 days	7.90^d^	8.83^b^	10.22^a^	8.18^c^	0.190	0.001	0.001	0.001
ADG (g/d)	77.67^b^	86.45^a^	88.52^a^	68.78^c^	1.982	0.001	0.034	0.001
DMI (g/d)	436.17^a^	434.50^a^	435.00^a^	423.17^b^	1.350	0.001	0.001	0.033
FCR (kg/kg)	5.67^a^	4.88^b^	4.95^b^	6.22^a^	0.151	0.001	0.086	0.001

a, b, c, d *Means with different superscripts in each row differ significantly (P < 0.05)*.

e *Control (without CO), LCO, MCO and HCO with 4, 6, and 8 g CO per goat per day, respectively*.

f *ADG, average daily bodyweight gain; DMI, dry matter intake; FCR, feed conversion ratio*.

g *SEM, standard error of the mean (n = 6)*.

### Nutrient Digestibility and Ruminal Fermentation Parameters

As presented in Table 4, the digestibility of crude protein (CP) was not affected by CO addition. The digestibility of DM, OM, aNDF and ADF decreased linearly (*P* < 0.05) with increasing CO supplementation, and was lower for MCO than that of control, LCO and HCO groups (*P* < 0.05). However, the digestibility of EE increased linearly (*P* < 0.05) and quadratically (*P* < 0.05) with increasing CO supplementation, and was higher for MCO and HCO than control and LCO (*P* < 0.05).

**Table 4:** Effects of coconut oil on nutrient digestibility and ruminal fermentation in goat kids.

	**Treatment^e^**					***P-*****value**		
**Item**	**CON**	**LCO**	**MCO**	**HCO**	**SEM^f^**	**Treatment**	**Linear**	**Quadratic**
**Nutrient digestibility (%)**								
Dry matter	0.63^a^	0.62^a^	0.62^a^	0.60^b^	0.003	0.001	0.001	0.121
Organic matter	0.62^a^	0.61^a^^b^	0.61^a^	0.59^b^	0.004	0.014	0.005	0.461
Crude protein	0.74	0.73	0.73	0.73	0.005	0.875	0.456	0.835
Ether extract	0.61^d^	0.66^c^	0.72^b^	0.79^a^	0.014	0.001	0.001	0.042
Neutral detergent fiber	0.57^a^	0.52^c^	0.54^b^	0.51^d^	0.005	0.001	0.001	0.301
Acid detergent fiber	0.43^a^	0.41^b^	0.41^b^	0.39^c^	0.005	0.001	0.001	0.775
Ruminal fermentation pH	6.35^b^	6.46^a^	6.44^a^^b^	6.48^a^	0.018	0.033	0.013	0.276
Total volatile fatty acid (mmol/L)	91.15^a^	90.16^a^	90.53^a^	88.01^b^	0.423	0.016	0.006	0.192
Mol/100 mol								
Acetate (A)	72.41^a^	70.74^a^	70.09^a^^b^	68.79^b^	0.491	0.011	0.002	0.464
Propionate (P)	20.94	20.31	20.00	20.34	0.188	0.385	0.234	0.221
Butyrate	13.84^a^	13.18^a^^b^	13.02^b^	11.77^c^	0.236	0.001	0.001	0.107
Valerate	1.74	1.72	1.72	1.70	0.008	0.547	0.221	0.789
Isobutyrate	1.12^a^	1.05^b^	1.07^b^	0.96^c^	0.021	0.020	0.005	0.527
Isovalerate	1.53^a^	1.46^a^^b^	1.50^a^	1.41^b^	0.017	0.032	0.016	0.833
Acetate/Propionate	3.47	3.46	3.50	3.38	0.024	0.457	0.349	0.320
Ammonia-N (mg/100 ml)	12.71^a^	12.50^a^^b^	12.39^a^^b^	12.13^b^	0.092	0.158	0.032	0.886
Methane (mol/mol TVFA)	32.04^a^	31.52^b^	31.29^b^	30.07^c^	0.224	0.001	0.001	0.012

a, b, c, d *Means with different superscripts in each row differ significantly (P < 0.05)*.

e *Control (without CO), LCO, MCO and HCO with 4, 6, and 8 g CO per goat per day, respectively*.

f *SEM, standard error of the mean (n = 6)*.

Furthermore, ruminal pH increased linearly (*P* < 0.05) with increasing CO supplementation and was higher for HCO and LCO than control (*P* < 0.05). Total ruminal VFA concentration linearly decreased (*P* < 0.05) and was lower for HCO group than other three groups (*P* < 0.05). The molar proportions of propionate, valerate, and the ratio of acetate to propionate were not affected (*P* > 0.05), but the molar proportions of acetate, butyrate, isobutyrate and isovalerate linearly (*P* < 0.05) decreased with increasing CO supplementation, and was lower for the HCO than for control, LCO and MCO (*P* < 0.05). Ruminal ammonia N content linearly reduced by increasing CO supplementation (*P* < 0.05). The estimated methane emission decreased linearly (*P* < 0.05) and quadratically (*P* < 0.05) with increasing CO supplementation and was lower for the LCO, MCO, and HCO than control (*P* < 0.05).

### Ruminal Microbial Enzyme Activity and Populations of Ruminal Cellulolytic Bacteria

The enzymatic activities of caboxymethyl-cellulase, cellobiase, xylanase, pectinase and α-amylase linearly (*P* < 0.05) decreased with increasing CO supplementation, and were lower for HCO than the control (*P* < 0.05) (Table 5).

**Table 5:** Effects of coconut oil on rumen microbial enzyme activity and ruminal microflora in goat kids.

	**Treatment^e^**					***P*****-value**		
**Item**	**CON**	**LCO**	**MCO**	**HCO**	**SEM^h^**	**Treatment**	**Linear**	**Quadratic**
**Microbial enzyme activity^f^**								
Caboxymethyl-cellulase	0.32^a^	0.28^b^	0.28^b^	0.23^c^	0.010	0.001	0.001	0.658
Cellobiase	0.14^a^	0.13^a^^b^	0.13^a^^b^	0.11^b^	0.004	0.129	0.041	0.420
Xylanase	0.45^a^	0.39^b^	0.39^b^	0.35^b^	0.012	0.011	0.002	0.559
Pectinase	0.33^a^	0.29^b^	0.24^c^	0.21^d^	0.014	0.001	0.001	0.688
α-amylase	1.56^a^	1.46^b^	1.46^b^	1.40^c^	0.019	0.001	0.001	0.343
Protease	0.47^a^	0.46^a^	0.43^b^	0.42^b^	0.007	0.001	0.001	0.706
**Microbiota (copies/ml)^g^**								
Total bacteria × 10^11^	1.77^a^	1.46^b^	1.44^b^	1.40^b^	0.046	0.001	0.001	0.001
protozoa × 10^5^	5.42^a^	4.69^b^	4.12^c^	2.71^d^	0.308	0.001	0.001	0.076
*R. albus* × 10^8^	0.71^a^	0.36^b^	0.35^b^	0.04^c^	0.079	0.002	0.001	0.816
*R. flavefaciens* × 10^8^	0.74^a^	0.32^b^	0.35^b^	0.09^c^	0.077	0.001	0.001	0.291
*F. succinogenes* × 10^8^	0.51^a^	0.49^a^	0.48^a^	0.15^b^	0.046	0.001	0.001	0.001
*B. fibrisolvens* × 10^8^	1.75^a^	1.58^b^	1.54^b^	1.39^c^	0.039	0.001	0.001	0.524
*P*. ruminicola × 10^8^	0.82^a^	0.41^b^^c^	0.37^b^^c^	0.20^c^	0.072	0.001	0.001	0.052
*R. amylophilus* × 10^9^	0.74^a^	0.38^b^	0.17^c^	0.03^d^	0.087	0.001	0.001	0.195

a, b, c, d *Means with different superscripts in each row differ significantly (P < 0.05)*.

e *Control (without CO), LCO, MCO and HCO with4, 6and 8 g CO per goat per day, respectively*.

f *Units of enzyme activity are: carboxymethyl cellulase (μmol glucose/min/ml), cellobiase (μmol glucose/min/ml), xylanase (μmol xylose/min/ml), pectinase (μmol D-galactouronic acid /min/ml), α-amylase (μmol glucose/min/ml) and protease (μg hydrolysed protein/min/ml)*.

g *R. albus, Ruminococcus albus; R. flavefaciens, Ruminococcus flacefaciens; B. fibrisolvens, Butyrivibrio fibrisolvens; F. succinogenes, Fibrobacter succinogenes; R. amylophilus, Ruminobacter amylophilus; P. ruminicola, Prevotella ruminicola*.

h *SEM, standard error of the mean (n = 6)*.

Total bacterial and *F. succinogenes* populations linearly (*P* < 0.05) and quadratically (*P* < 0.05) decreased, and the populations of *R. albus, R. flavefaciens, B. fibrisolvens, P. ruminicola*, and *R. amylophilus* decreased linearly (*P* < 0.05) with increasing CO supplementation (Table 5).

#### Blood Metabolites

The serum concentration of TGs, NEFAs, and GH linearly (*P* < 0.05) increased with increasing CO supplementation and was higher for HCO group than for the control (*P* < 0.05). Nevertheless, serum glucose and TC were not affected by CO supplementation (*P* > 0.05) for pre-weaning and post-weaning goats (Table 6).

**Table 6:** Effects of coconut oil on serum biochemical indices and hormone secretion in goat kids.

	**Treatment^c^**					***P*****-value**		
**Item^d^**	**CON**	**LCO**	**MCO**	**HCO**	**SEM^e^**	**Treatment**	**Linear**	**Quadratic**
**70 days of age**								
TC (mmol/L)	3.69	3.49	3.86	4.20	0.174	0.571	0.261	0.468
TG (mmol/L)	0.58^b^	0.67^a^^b^	0.75^a^^b^	0.82^a^	0.045	0.136	0.024	0.830
Glucose (mmol/L)	4.50	3.85	3.99	4.08	0.113	0.155	0.226	0.077
NEFA (mmol/L)	0.19^b^	0.21^b^	0.27^a^^b^	0.32^a^	0.020	0.037	0.007	0.560
GH (ug/L)	5.60^b^	6.59^a^^b^	7.47^a^	6.82^a^^b^	0.267	0.067	0.040	0.086
**100 days of age**								
TC (mmol/L)	3.77	3.72	3.94	4.31	0.162	0.217	0.070	0.316
TG (mmol/L)	0.65^b^	0.72^b^	0.79^a^^b^	0.90^a^	0.034	0.027	0.004	0.679
Glucose (mmol/L)	4.62	4.43	4.28	4.23	0.087	0.285	0.089	0.485
NEFA (mmol/L)	0.25^b^	0.29^a^^b^	0.32^a^^b^	0.38^a^	0.019	0.081	0.015	0.751
GH (ug/L)	6.41^b^	6.94^b^	7.68^a^	7.04^a^^b^	0.161	0.014	0.019	0.028

a, b *Means with different superscripts in each row differ significantly (P < 0.05)*.

c *Control (without CO), LCO, MCO and HCO with4, 6, and 8 g CO per goat per day, respectively*.

d *TC, Total-cholestero; NEFA, non-esterified fatty acid; TG, triacylglycerols; GH, Growth hormone*.

e *SEM, standard error of the mean (n = 6)*.

## Discussion

### Growth Performance of Goat Kids

An appropriate amount of energy supply is the key to ensure and promote the healthy and rapid growth and development of young ruminants. Dietary MCFA (medium chain fatty acid) can effectively reduce body fat deposition and improve lipid concentration. However, there have been discrepancies in the results of coconut oil supplementation on nutrient digestibility, growth performance, etc. The decrease in DM intake with increasing CO supplementation was consistent with the findings of other studies, in which DM intake was decreased by CO supplementation (25, 50, and 75 g/kg of concentrate) in the diet of lambs (10). This reduced DM intake was not surprising, since the negative effects of CO on DMI may be a consequence of higher energy density in the diet (25, 26). Moreover, higher CO inclusion have been shown to be related to decreased NDF digestion (27) and palatability (28). Hollmann and Beede (29) also reported that CO replacement of ground corn in the diets of lactating dairy cows with CO lead to a significant reduction in the DMI. Linear and quadratic increments in the ADG with higher CO supplementation were observed in our experiment. Meanwhile, FCR in this study quadratically decreased with CO supplementation during the pre-weaning and post-weaning periods. This could be attributed to the improvement in the energy intake level and EE digestibility by CO supplementation, essentially due to the higher EE levels of CO-supplemented diets. Similarly, Dutta et al. (30) reported a gradual increase in ADG up to 50 g/kg fat supplementation, but above this level it declined. Unlike our finding, Bhatt et al. (10) reported that increasing CO supplementation had no effect on ADG of lambs during the pre-weaning and post-weaning periods, which might be due to the heat stress in their study.

### Nutrient Digestibility

Adding appropriate fat or fatty acid into ruminates can promote nutrient digestibility (8). The linear EE digestibility increments with higher CO supplementation observed in this study were also reported by Bhatt et al. (10). No change was observed in CP digestibility, but DM, OM, ADF, and NDF digestibility linearly decreased when increasing CO feeding portions. Similar results have been reported after CO addition (29, 31). This phenomenon could be the result of several factors. For instance, CO supplementation could markedly reduce the number of rumen protozoa (10). Rumen protozoa exhibited cellulase, hemicellulase, and pectinase activities (32–34), which may explain their role in NDF digestion. Additionally, rumen protozoa may also alter the number of cellulolytic bacteria, and thus affect the extent of ruminal fiber fermentation (35). In the present study, the reduction in NDF digestibility was consistent with a reduction in protozoal numbers by increasing CO supplementation, and this maybe a reason for reduced NDF and ADF digestibility by increasing CO feeding portions.

### Ruminal Fermentation, Microorganism Population and Enzyme Activities

Fat can be used as carrier of fat-soluble vitamins and promote the absorption and utilization of fat-soluble vitamins, negative effects on rumen microbes, fiber digestion, and fermentation (10, 14). The pH in rumen fluid linearly increased with increasing CO supplementation and was higher for LCO and HCO than MCO and control group, which was similar with the observation of Pilajun et al. (36), who reported that ruminal pH was directly proportional to the dosage of CO replacing sunflower oil from 250 to 750 g/kg in steers. Ruminal microbes can utilize ruminal ammonia-N derived from protein degradation for microbial protein synthesis (37). The lower ammonia-N levels produced by goats receiving CO supplementation were likely due to decreased protease activity, and reduced rumen bacterial and protozoal populations.

CO supplementation linearly decreased the total VFA concentration in the rumen. The results of this study were consistent with those obtained by Machmüller et al. (38), who found that CO supplementation tended to decrease the total VFA concentration. This finding could be due to the inhibitory effect of fatty acids on fiber digestion (39) and toxicity of fats to microorganisms (40). Moreover, no differences were observed in the molar proportion of propionate, but the molar proportions of acetate and butyrate were decreased with higher levels of CO supplementation. The possible explanation for this phenomenon was that CO inhibited bacteria and protozoa that are not related to *Selenomonas ruminantium*, which is essential to propionate production (41). Ruminal cellulolytic bacteria and protozoa produce cellulolytic enzymes and degrade dietary fiber to acetate (42). Thus, the lower acetate molar proportion resulted from the decrease in activity of carboxymethyl-cellulase, cellobiase and xylanase as well as the total population of bacteria, protozoa, and cellulolytic bacteria (*R. albus, R. flavefaciens, B. fibrisolvens*, and *F. succinogenes*) following CO addition. Being a by-product of carbohydrate fermentation, butyrate is produced by ruminal protozoa (43). Similarly, Hristov et al. (41) reported that CO supplementation inhibited both protozoa and important butyrate producers in the rumen, such as *B. fibrisolvens*. Hence, the decrease in the population of protozoa with CO supplementation observed in the present study also provides evidence for the reduction in the molar proportion of butyrate. In agreement with this study, similar findings in total VFA concentration (38), proportion of acetate (17), propionate (17), and A:P ratio (17) were reported in other previous studies. In contrast, Bozzolo et al. (44) found that dietary supplementation of 50 g/kg of CO had no significant effect on the concentration of VFA in the rumen of lambs for a period of 2 weeks directly after weaning. The inconsistency in these results could be due to that in their experiment, the lambs among the treatment fed the same level of fatty acids included in the diet, whereas in our experiment, goat kids in each treatment were fed a diet with different levels of total energy intake.

Calculation of ruminal methane production using VFAs based on this study's procedure demonstrated that CO supplementation elicited a significantly linear and quadratic decline in methane production. The protozoa populations were also linearly reduced by CO addition. These results were consistent with those of *in vitro* (45) and *in vivo* studies (15, 28), which have confirmed the methane-suppressing effect of CO supplementation in ruminants.

The linear decrease in the total population of bacteria, protozoa, *R. amylophilus*, and predominant cellulolytic bacteria (*R. albus, R. flavefaciens, B. fibrisolvens, and F. succinogenes*) with increasing CO supplementation suggested that CO modulates the ruminal microorganisms in a dose-dependent manner. The toxicity of Medium-chain saturated FAs to the ruminal microbiota has been well-documented. Work by Hristov et al. (41) has confirmed that CO supplementation results in statistically significant suppression of microbial flow. Inhibition of total bacterial counts, cellulolytic and amylolytic species secondary to CO administration was reported by Dong et al. (45). *In vitro* study carried out by Patra and Yu (8) also reported that CO exerted inhibitory effects on protozoa and cellulolytic bacteria (*F. succinogenes* and *R. flavefaciens*). This decrease might be explained by the inhibitory effect of CO on protozoa or certain bacteria species that suppress the growth of cellulolytic bacteria in the rumen. The linear decrease in NDF digestibility with CO supplementation also provides evidence for the potential inhibitory effects of CO on rumen cellulolytic bacteria.

Rumen enzyme activity is closely related to the growth status of ruminal bacteria and then affects the degradation ability to nutrient (46). In the present study, the linear decrease in the enzymatic activities of caboxymethyl-cellulase, cellobiase, xylanase, pectinase, α-amylase, and protease with increasing CO supplementation confirmed the modulation of ruminal microbial activity by CO. Additionally, the decreased enzymatic activities of caboxymethyl-cellulase, cellobiase, xylanase, and pectinase were primarily attributed to the suppression of cellulolytic bacteria growth, hence resulting in a decreased NDF and ADF digestibility. *P. ruminicola* and *R. amylophilus* are able to secrete large amounts of α-amylase (47). The linear decrease in the enzymatic activities of α-amylase noticed in this study coincided with the decrease in the total number of *P. ruminicola* and *R. amylophilus* with increasing CO supplementation. In addition, the linear decrease in protease enzymatic activities was related to the inhibitory effect of CO on proteolytic bacteria. This finding was supported by the decreased ruminal ammonia-N concentration and CP digestibility.

### Serum Biochemical Parameters

The serum concentration of glucose and TC were not affected by the treatments. In contrast, studies conducted in finishing heifers (48) and lambs (10) found an increase in serum cholesterol levels following CO supplementation. The discrepancy is attributed to the difference in animals in these studies. However, the serum concentration of TGs and NEFAs linearly increased with augmentation of CO supplementation. Circulating NEFAs derived from digestive tract absorption and adipose tissue release could be used to reflect the mobilization of body fat and metabolism of fatty acids (49, 50). In the present study, the higher blood concentrations of NEFA in HCO supplementation reflected the promoting of body fat mobilization as indicated by negative BW changes compared with the goats in MCO group. Furthermore, serum concentrations of GH are affected by the nutrient level and growth performance (51). In this present study, serum concentrations of GH exhibited a linear increase with increments in CO supplementation. This supports the hypothesis that optimum CO supplementation could result in positive responses of serum GH concentration and improvement of the goat kids' growth performance.

## Conclusion

In summary, CO supplementation at 6 g/day per goats is optimum in goat kids due to improved ADG and feed conversion efficiency and decreased estimated methane emission. Supplementation CO up to 8 g/day depressed growth and feed conversion due to its suppression of growth performance, rumen protozoa, cellulolytic bacteria (*R. albus, R. flavefaciens, B. fibrisolvens*, and *F. succinogenes*) and microbial enzyme activity (caboxymethyl-cellulase, cellobiase, xylanase, pectinase, α-amylase, and protease), and reduced ADF and ADF digestibility.

## Data Availability Statement

The original contributions presented in the study are included in the article/Supplementary Materials, further inquiries can be directed to the Corresponding authors.

## Ethics Statement

The animal study was reviewed and approved by Committee on laboratory animal ethics of Tropical Crops Genetic Resources Institute (TCGRI). Written informed consent was obtained from the owners for the participation of their animals in this study.

## Author Contributions

LS, WX, and QL designed the experiment. LS, YZ, and LW conducted the experiment. YZ, TC, and GH collected and analyzed data. LS and WX prepared the manuscript. All authors contributed to the article and approved the submitted version.

## Conflict of Interest

The authors declare that the research was conducted in the absence of any commercial or financial relationships that could be construed as a potential conflict of interest.

## Abbreviations

ADF: acid detergent fiber
ADG: average daily gain
BW: body weight
CO: coconut oil
CP: crude protein
DM: dry matter
DMI: dry matter intake
average daily feed intake: ADFI
EE: ether extract
FCR: Feed conversion ratio
GH: growth hormone
NDF: neutral detergent fiber
NEFA: non-esterified fatty acid
OM: organic matter
RT-PCR: real time polymerase chain reaction
TG: triglyceride
VFA: volatile fatty acids.
